# The association between physical performance and subjective wellbeing in Chinese older adults: A cross-sectional study

**DOI:** 10.3389/fpubh.2022.965460

**Published:** 2022-09-15

**Authors:** Haiyang Xie, Shenghua Lu

**Affiliations:** ^1^Yong In University, Yongin-si, South Korea; ^2^College of Sports Science, Jishou University, Jishou, China; ^3^Hunan Academy of Education Sciences, Changsha, China

**Keywords:** handgrip strength, gait, wellbeing, Chinese older adults, quality of life

## Abstract

**Purpose:**

This study aimed to investigate the association between physical performance and subjective wellbeing among Chinese older adults.

**Methods:**

Data on the Chinese population were gathered from the Study on Global Aging and Adult Health Survey (SAGE). This survey used a stratified multistage cluster sample design based on geographical location and economic status. Chinese older adults aged 65 years old or above from eight provinces (Guangdong, Hubei, Jilin, Shaanxi, Shandong, Shanghai, Yunnan, and Zhejiang) were included in this cross-sectional study. Physical performance was measured using relative handgrip strength and normal gait speed. Subjective wellbeing was measured using quality-of-life (QOL), happiness, and mood through interviews with participants. Logistic regressions were used to examine the associations between physical performance and each of the three wellbeing variables (QOL, happiness, and mood).

**Results:**

Data of 5,421 Chinese older adults (mean age: 72.93 ± 5.89 years old, 47.1% men) were analyzed. In this sample, individuals with a higher level of relative handgrip strength (rHGS) had better mood compared to those with a lower level of rHGS (*p* < 0.05), and persons with lower gait speed had poorer QOL, happiness, and mood compared to those with faster gait speed (*p* < 0.05).

**Conclusion:**

Our findings suggest that a higher level of relative handgrip strength predicted better mood and lower gait speed predicted poor QOL, happiness, and mood in Chinese older adults.

## Introduction

China has the fastest-growing aging population worldwide. With longer life expectancy and reduced fertility rates, 366 million Chinese people will be aged 65 years old or over by 2050 (1). Such significant changes have not only produced multiple challenges for many countries globally and economically but also place substantial pressures on almost every household. Furthermore, managing this rapidly growing number of older adults is more challenging for China as it is a developing country with one of the largest populations in the world and has a relatively limited healthcare system to care for its aging population compared with western developed countries and more developed regions (2). Many studies have shown that the greatest proportion of chronic illnesses occurs among older adults, and this has been accepted as a universal problem that requires special attention (1, 3).

Normal aging is closely linked to reduced subjective wellbeing (defined as physically and mentally subjective perceptions about daily activities) (4, 5). For example, reduced QOL, unhappiness, and mood dysfunction (measured by using a self-reported questionnaire) are commonly reported health issues resulting from aging. In contrast, individuals with better moods have a lower risk of disabilities, fewer symptoms, and fewer complaints of pain in later life (6). Thus, those with high levels of subjective wellbeing tend to have healthier lives than people with low levels of subjective wellbeing. To effectively improve subjective wellbeing, it is crucial to identify protective factors that can help older adults to live well.

Existing evidence indicates that physical activity is associated with physical and psychological wellbeing in older individuals. For example, regularly engaging in physical activity is significantly associated with better physical function (7), decreased severe mental health, and improved subjective wellbeing in older people (8, 9). Review studies have supported the beneficial effects of engagement in physical activity on the different aspects of psychological wellbeing among the older population (10–12). However, the association between physical function and psychological wellbeing has not been established, and physical function may be crucial for promoting psychological wellbeing in older people (13).

Physical function is the ability to carry out the basic, instrumental activities of daily life (13). In recent years, a substantial number of studies have focused on the physical performance of older adults as they face natural functional decline and sought to determine whether physical measures (e.g., handgrip strength and gait speed) are independent predictors of multiple health-related issues (14–16). For example, regardless of gender, aged individuals with low relative handgrip strength (rHGS) were reported to have reduced QOL (17, 18), a greater degree of depressive symptoms (19), and a lower level of self-perceived happiness (20).

In addition, compared with rHGS, gait speed as an essential component of physical function should receive greater attention, especially in establishing a link between the indicators of functional performance and subjective wellbeing in older adults. Moreover, declined gait speed may negatively influence QOL by accelerating dependency on others to carry out daily activities, increasing the risk of disabilities and hospitalization (21–23), and affecting wellbeing. For example, experiencing negative emotions (e.g., anxiety and depression) is closely associated with declines in physical function (e.g., gait speed) in western populations (24–26). Feldman et al. found that individuals with slower gait speed likely have anxiety symptoms (27). Accordingly, preserving a high gait speed can enhance mental health and wellbeing among senior adults (28, 29).

Collectively, the psychological health benefits of maintaining effective physical function have been widely studied in western countries. However, the link between physical function and subjective wellbeing is less well understood. Only one study investigated the association between physical function and wellbeing in low- and middle-income countries, reporting that higher levels of physical function were associated with better wellbeing in adults (16). However, it is unclear whether the link exists among Chinese seniors. Therefore, this study aimed to investigate the associations between physical performance and subjective wellbeing in Chinese over 65 years of age. It is hoped that the results will help health professionals and clinicians to better design behavioral intervention programs for older adults to maintain better wellbeing as they age.

## Methods

### Participants

Data from a cross-sectional survey by the SAGE (https://apps.who.int/healthinfo/systems/surveydata/index.php/catalog/sage) was used to investigate the association between gait speed, rHGS, and subjective wellbeing in Chinese older adults. This survey used a stratified multistage cluster sample design based on geographical location and economic status. It is a nationally representative sample of populations from eight Chinese provinces (Guangdong, Hubei, Jilin, Shaanxi, Shandong, Shanghai, Yunnan, and Zhejiang). A total of 5,421 individuals aged 65 years or older were included in the current study after excluding individuals with incomplete data. The entire data collection approach has been described in a previous study (30). The participants completed the interview questionnaires under the guidance of the interviewers. The survey response rate was 93%. This study complies with the Declaration of Helsinki and was granted ethical approval by the Chinese ethics research review board at Peking University. All participants agreed to the experimental conditions and signed an informed consent form.

### Handgrip strength

To measure isometric rHGS with a handhold Smedley's dynamometer (Scandidact, Denmark), the participants were seated in a comfortable chair with their feet flat on the ground. They were asked to keep the shoulder of the tested arm in an adducted and neutrally rotated position with the elbow flexed at 90 degrees. Furthermore, during the assessment, the participants were instructed to maintain a neutral wrist position (thumb facing upward). We asked participants to squeeze their hands as hard as they could for 3 s to gain the maximal handgrip strength of each hand (left hand and right hand). All participants conducted the tests two times taking each side in turn. The average sum of the best results achieved with each hand was deemed to be the overall handgrip strength. The value of rHGS was calculated by dividing handgrip strength by body mass index (BMI) as described in a previous study (31).

### Gait speed

To assess gait speed with a timer, the participants were asked to walk at a normal speed over a 4-m flat area. Walk time was recorded using a stopwatch, and the gait speed was calculated by dividing the distance by the walk time for participants who did not have disabling conditions. The relative gait speed was expressed using the gait speed divided by height (in meters) as described in a previous study (32).

### Subjective wellbeing

The SAGE questionnaire on subjective wellbeing includes 10 items based on the World Mental Health Survey. All questions were translated into Chinese. Previous researchers have suggested that wellbeing studies should include several variables to provide a comprehensive understanding of mental health (33) and capture the attitudes and opinions of participants with minimum bias (34). Therefore, in this study, subjective wellbeing was measured using three common question items, in line with a recent study (35): (1) QOL was tested using the item: *How would you rate your overall QOL?;* the response options were: *very good, good, moderate, bad, and very poor*. The responses were recoded as dichotomous QOL variables, of which, a rating of *very good* and *good* was classified as *good QOL*; a rating of *very poor, poor*, or *moderate* was classified as *poor QOL*. (2) Happiness was tested using the item: *Taking all things together, how would you say you are these days?*; the response options were: *very happy,” happy, neither happy nor unhappy, unhappy*, and *very unhappy*. The responses were recoded as dichotomous happiness variables, of which, a rating of *very happy* and *happy* was classified as *good*; a rating of *very unhappy, unhappy*, or *neither happy nor unhappy* was classified as *unhappy*. (3) Mood was tested using the item: *Are you usually in a better mood or a worse mood than most others? Or are you about the same?;* the response options were: *better, the same*, and *worse*. These responses were recoded as dichotomous mood variables, of which, a rating of *better* was classified as *good mood*; a rating of *the same* or *worse* was classified as *poor mood*. The same method of categorizing the responses to subjective wellbeing items was used as that used in the previous study (35).

### Control variables

The control variables were age, sex, years of education, setting (rural or urban), smoking status, alcohol consumption, number of chronic diseases (e.g., arthritis, stroke, and diabetes) (36), and BMI. Years of education were classified as 0 (less than primary school), 1–6 (completed primary school), 7–12 (completed secondary school or high-school equivalent), and >12 (completed college or post-graduate degree). Smoking status was classified as currently smoking, ex-smoker, and never smoked. Alcohol consumption was self-reported based on alcohol consumed in the last month. The number of chronic diseases was calculated by adding up the number of self-reported diagnosed diseases. The BMI was calculated by dividing weight (in kilograms) by height^2^ (in meters).

### Statistical analysis

The statistical analyses were conducted using Stata 15.0 (Stata Corp LP, College Station, Texas). The level of statistical significance was set at *p* < 0.05. The descriptive characteristics were summarized and presented as mean and standard deviations (SDs), totals, and percentages. To examine the association between rHGS and the dependent variables, a binary logistic regression model was used. We then examined the relationships among rHGS, QOL, happiness, and mood using binary logistic regression after controlling for the impacts of the control variables, respectively. The results were presented as an odds ratio (*OR*) with a 95% confidence interval (*CI*).

## Results

The analytical sample consisted of 5,421 adults aged 65 years old or above (Table 1). In this sample, the mean age was 72.93 ± 5.89 years, 47.1% were male participants, 15.9% of participants had no educational experience, and 55% of participants were living in urban areas. The proportions of current smokers and those who consumed alcohol were 69.3 and 26.6%, respectively. The proportion of those who were disease-free was 35.5%. The mean handgrip strength and gait speed were 23.0 ± 11.3 kg and 4.98 ± 1.8 s, respectively. The proportions of those with good QOL, happiness, and good mood were 32.9, 55.0, and 16.2%, respectively.

**Table 1:** Sample characteristics.

**Variables**		***n***	**Mean ±SD/%**
Age		5,421	72.93 ± 5.89
Gender	Male	2,553	47.1
	Female	2,868	52.9
Education (years)	0	862	15.9
	1–6	1,556	28.7
	7–12	509	9.4
	>12	314	5.8
	Missing	2,180	40.2
Setting	Rural	2,442	45.0
	Urban	2,979	55.0
Alcohol consumption	Yes	1,443	26.6
	No	3,759	69.3
	Missing	219	4.1
Smoking	Never	3,567	65.8
	Current	1,168	21.5
	Past	464	8.6
	Missing	222	4.1
Number of chronic diseases	0	1,926	35.5
	1–2	2,601	48.0
	≥3	572	10.6
	Missing	322	5.9
Handgrip strength (kg)		4,809	23.0 ± 11.3
	Missing	612	
Gait speed(s)		4,826	4.98 ± 1.8
	Missing	595	
Quality of life	Good	1,782	32.9
	Poor	3,350	61.8
	Missing	289	5.3
Happiness	Yes	2,981	55.0
	No	2,135	39.4
	Missing	305	5.6
Mood	Good	877	16.2
	Poor	4,304	79.4
	Missing	240	4.4

Table 2 shows the bivariate association between physical performance (rHGS and gait speed) and wellbeing (QOL, happiness, and mood) in the participants. Individuals with the highest tertile of rHGS (*OR* = 1.46, *p* = 0.01) and moderate rHGS (*OR* = 1.48, *p* = 0.004) had good moods compared to those with the lowest tertile of rHGS. With respect to the gait speed, individuals with the lowest tertile of gait speed had lower QOL (*OR* = 0.71, *p* = 0.002), more unhappiness (*OR* = 0.62, *p* < 0.01), and bad mood (*OR* = 0.69, *p* = 0.003) compared to those with the highest tertile of gait speed.

**Table 2:** The association between physical performance and wellbeing estimated by logistic regression models.

	**Quality of life**				**Happiness**				**Mood**			
	***p***	**OR**	**95%CI**		***p***	**OR**	**95%CI**		***p***	**OR**	**95%CI**	
Age	0.00	1.03	1.01	1.05	0.003	1.03	1.01	1.04	0.11	1.02	1.00	1.04
Sex	0.98	1.00	0.80	1.24	0.77	1.03	0.83	1.29	0.71	1.05	0.81	1.35
Education (years)												
0	Reference	Reference	Reference									
1–6	0.11	1.18	0.96	1.45	0.83	1.02	0.84	1.25	0.46	1.10	0.86	1.40
7–12	0.00	1.88	1.43	2.47	0.20	1.21	0.91	1.60	0.01	1.49	1.09	2.05
>12	0.00	2.24	1.61	3.10	0.26	1.22	0.87	1.71	0.001	1.91	1.32	2.75
Handgrip strength												
Low	Reference	Reference	Reference									
Moderate	0.10	0.83	0.66	1.02	0.051	0.81	0.64	1.00	0.004	1.48	1.13	1.94
High	0.39	0.90	0.71	1.14	0.61	1.07	0.84	1.36	0.01	1.46	1.09	1.95
**Gait speed**												
Low	0.002	0.71	0.57	0.88	0.00	0.62	0.50	0.77	0.003	0.69	0.54	0.88
Moderate	0.07	0.82	0.66	1.02	0.09	0.83	0.66	1.03	0.06	0.80	0.63	1.01
High	Reference	Reference	Reference									

OR, odds ratio; CI, confidence interval.

The adjusted model included age, sex, education years, setting, alcohol consumption, smoking, number of chronic diseases, and body mass index (BMI).

## Discussion

This cross-sectional study examined the association between physical performance and subjective wellbeing among Chinese seniors. The findings suggested that a higher level of rHGS was significantly associated with better mood through controlling for the covariates (e.g., age and years of education); the lower gait speed was significantly associated with poor wellbeing in terms of QOL, happiness, and mood.

Handgrip strength is a proxy measurement for overall strength, predicting many health outcomes. In this study, older adults with a higher level of rHGS had a significantly better mood. This finding is in accordance with previous works (37, 38). An association between HGS and mood was investigated by Laredo-Aguilera et al. (39). This association may be attributed to the fact that a higher HGS represented complete physical function, which is the most essential requirement for performing daily activities with ease. Thereby, the person with higher rHGS can finish all daily activities that they can, and get in a better mood (39). Even so, there was no significant association between rHGS and QOL and happiness. These findings were inconsistent with other studies (40, 41). Relatedly, Garatachea et al. investigated whether physical function was related to subjective wellbeing in Spanish seniors (13) and found that HGS was significantly correlated with QOL. Moreover, positive relationships between HGS and QOL and happiness were observed in rural Chinese populations (15). These inconsistent results may be due to the differences in measuring instruments and measurement methods. In the current study, participants carried out the HGS testing in a sitting posture, keeping the shoulder of the tested arm in an adducted and neutrally rotated position with the elbow flexed at 90 degrees, whereas in (38), participants were in a standing posture with fully extended their arm, forming an angle of 30 degrees to their trunk. Further studies could verify this association between HGS and wellbeing in Chinese older adults.

Gait speed is a valid and reliable measure for assessing and monitoring physical function and psychological health status (42). The findings of the current study suggested that older adults with lower gait speeds were more likely to report poor wellbeing in QOL, happiness, and mood. Although many factors can influence wellbeing, previous studies have suggested a close association between gait speed and wellbeing, which concurs with the results of the present study (43–45). Kim et al. investigated the relationship between gait pattern and emotional state by utilizing virtual reality technology (46). They reported that a slower gait speed was significantly linked to poor emotional states (a lack of happiness and good mood) in healthy adults. In contrast, a faster gait speed was correlated with higher health-related QOL scores (44). Moreover, gait pattern is controlled by psychomotor skills, which are easily influenced by negative emotions or poor wellbeing (e.g., depression and unhappiness) (46). Thus, it is likely that a slower gait speed can predict poor wellbeing. These results help to explain our findings that Chinese older adults with slower gait speeds had significantly poorer subjective wellbeing.

Given the rapidly increasing older population and the risk of disease and mental health problems, identifying effective interventions is particularly important. The current study's findings suggest that improving physical function (e.g., gait speed and rHGS) may benefit subjective wellbeing, at least in Chinese seniors. In the meantime, the results also imply that engagement in physical activity is an effective way to improve physical function and psychological wellbeing. Furthermore, clinicians should be aware of the association between physical function and subjective wellbeing and encourage older patients to adopt healthy lifestyles to improve their wellbeing. Further studies should explore the mechanisms that link physical function and subjective wellbeing.

Despite its merits, the present study is subjected to several limitations. First, the study's design was not intended to determine causality: whether physical performance was the causative reason for poor wellbeing in this sample. Second, the self-reported measures used to assess wellbeing as well as the other variables only indicate the subjects' mental health condition at a single point time across their entire life trajectory. Third, the sample size of each dependent variable may have restricted statistical power and affected the accuracy of the relationship between physical performance and wellbeing.

## Conclusion

The results of this cross-sectional study provided evidence of the association between physical performance and subjective wellbeing among Chinese older adults, suggesting that a higher level of rHGS was associated with better mood, and lower gait speed was associated with poor wellbeing in QOL, happiness, and mood.

## Data availability statement

The original contributions presented in the study are included in the article/supplementary material, further inquiries can be directed to the corresponding author/s.

## Ethics statement

The studies involving human participants were reviewed and approved by Chinese Ethics Research Review Board. The patients/participants provided their written informed consent to participate in this study.

## Author contributions

HX and SL: conceptualization and methodology. HX: data curation and analysis and writing the original draft preparation. SL: writing—review and editing. Both authors contributed to the article and approved the submitted version.

## Funding

This project was supported by the Key Research Project of the Hunan Educational Bureau (19A407).

## Conflict of interest

The authors declare that the research was conducted in the absence of any commercial or financial relationships that could be construed as a potential conflict of interest.

## Publisher's note

All claims expressed in this article are solely those of the authors and do not necessarily represent those of their affiliated organizations, or those of the publisher, the editors and the reviewers. Any product that may be evaluated in this article, or claim that may be made by its manufacturer, is not guaranteed or endorsed by the publisher.
